# Mixed Pulmonary Adenocarcinoma and Atypical Carcinoid: A Report of Two Cases of a Non-codified Entity With Biological Profile

**DOI:** 10.3389/fmolb.2021.784876

**Published:** 2021-12-03

**Authors:** Paola Parente, Antonio Rossi, Angelo Sparaneo, Federico Pio Fabrizio, Antonella Centonza, Marco Taurchini, Tommaso Mazza, Maurizio Cassano, Giuseppe Miscio, Flavia Centra, Gian Maria Ferretti, Concetta Martina Di Micco, Paolo Graziano, Lucia Anna Muscarella

**Affiliations:** ^1^ Unit of Pathology, Fondazione IRCCS Ospedale Casa Sollievo della Sofferenza, San Giovanni Rotondo, Italy; ^2^ Unit of Oncology, Fondazione IRCCS Ospedale Casa Sollievo della Sofferenza, San Giovanni Rotondo, Italy; ^3^ Laboratory of Oncology, Fondazione IRCCS Ospedale Casa Sollievo della Sofferenza, San Giovanni Rotondo, Italy; ^4^ Surgical Thoracic Unit, Fondazione IRCCS Ospedale Casa Sollievo della Sofferenza, San Giovanni Rotondo, Italy; ^5^ Bioinformatics Unit, Fondazione IRCCS Ospedale Casa Sollievo della Sofferenza, San Giovanni Rotondo, Italy

**Keywords:** lung, atypical carcinoid, adenocarcinoma, mixed neoplasm, monoclonality, next-generation sequencing

## Abstract

Pulmonary carcinoids combined with a non-neuroendocrine component have rarely been described, and this histological subtype is not included as a specific entity in the current World Health Organization classification of pulmonary neoplasms. Here, we described the molecular and histological features of two rare cases of mixed lung neoplasms, composed of atypical carcinoid and adenocarcinoma. The targeted next-generation sequencing analysis covering single nucleotide variations, copy number variations, and transcript fusions in a total of 161 cancer genes of the two different tumor components shows a similar molecular profile of shared and private gene mutations. These findings suggest their monoclonal origin from a transformed stem/progenitor tumor cell, which acquires a divergent differentiation during its development and progression and accumulates novel, specific mutations.

## Introduction

Combined or collision pulmonary tumors are primary lung neoplasms with two or more histologically distinct phenotypes (Olofson and Tafe, 2018). The World Health Organization (WHO) classification of the Tumors of the lung recognizes combined malignancies among neuroendocrine carcinomas (e.g., small cell lung cancer (SCLC) with large cell neuroendocrine lung cancer (LCNEC)] and combined SCLC or LCNEC with non-small cell lung cancer (NSCLC) histotype (WHO, 2021). Nevertheless, rare cases of typical and atypical carcinoids associated with squamous cell carcinoma or adenocarcinoma have been reported and extensively profiled at the molecular level (La Rosa et al., 2018). These combinations are not included as a specific entity in the WHO classification (WHO, 2021), and the pathogenesis of mixed histology is not well elucidated due to the rarity of presentation. The first proposed theory suggested a polyclonal origin with two independent precursors harboring different neoplasms (collision tumors). The second and more supported hypothesis indicates a monoclonal origin with a divergent differentiation of the two phenotypes starting from the same precursor clone (combined tumors) (La Rosa et al., 2018; Olofson and Tafe, 2018).

To date, only two cases of pulmonary adenocarcinoma (PA) mixed with atypical carcinoid (AC) with a detailed biological profile have been reported, and a monoclonal origin of the two components from a common transformed stem/progenitor tumor cell, which acquired divergent differentiation during neoplastic development, was suggested (i.e., epithelial carcinomatous, high grade, and epithelial neuroendocrine, low grade) (La Rosa et al., 2018; Olofson and Tafe, 2018). In both cases, the two components shared mutations in some genes, and additional mutations unique for each component were described in one case (La Rosa et al., 2018). However, the rarity of these tumors affects their deep and complete biological knowledge and the possibility to set an adequate medical therapy in metastatic/advanced patients.

Here, we present two novel additional cases of mixed PA and AC that were also profiled at the genetic level using next-generation sequencing (NGS) with a large gene panel including single nucleotide variations (SNVs), copy number variations (CNVs), and gene fusions.

## Material and Methods

### Patients Selection

Both patients presented as Case 1 and Case 2 underwent curative surgery at Istituto di Ricovero e Cura a Carattere Scientifico (IRCCS) Casa Sollievo della Sofferenza Hospital (San Giovanni Rotondo, FG, Italy). The clinical–pathological information and the biological material used in this study were collected following the Declaration of Helsinki, after the Local Ethics Committee Approval and with the informed consent of patients for genetic analysis of the lesions.

### Pathological Evaluation

Surgical specimens were examined and processed according to the current College of American Pathologists (CAP) guidelines. The specimens were fixed in 10% buffered formalin for 24–48 h, sampled and embedded in paraffin. Three-micrometer-thick tissue sections were cut and stained with hematoxylin and eosin (H&E). Immunohistochemical staining of sections representative of both neoplastic histotypes of primitive tumors and metastasis was carried out with the following antibodies: chromogranin A, synaptophysin, transcriptional thyroid factor-1 (TTF1, clone 8G7G3/1; Dako, Glostrup, Denmark), pan-cytokeratins (AE1-3 clone, Dako), cytokeratin 7, anaplastic lymphoma kinase (ALK, D5F3 clone, Ventana Medical Systems, Inc.), c-ROS oncogene 1 (ROS1, D4D6 clone, Cell Signaling), and tumor proportion score (TPS) of programmed death-ligand 1 (PDL1, clone 22C3, Dako) were also recorded based on recent guidelines.

### Molecular Biology

Unstained formalin fixed-paraffin embedded (FFPE) cancer tissue sections were microdissected to enrich for at least 60% neoplastic cellularity of each histological component of tumor. DNA and RNA were extracted using the QIamp DNA Micro kit (Qiagen) and RecoverAll™ Total Nucleic Acid Isolation kit (Ambion), respectively, and quantified with the Qubit fluorimeter (Thermo Fisher Scientific, Waltham, MA, United States). Relevant SNVs, CNVs, and gene fusions from a total of 161 cancer driver genes were searched using targeted NGS with Oncomine™ Comprehensive Assay v3M panel (Thermo Fisher, Life technologies Inc. division) and are listed in SupplementaryTable S1. Amplifications were performed starting from 30 ng of DNA/RNA, and libraries were run on GeneStudio S5. Raw signal data were analyzed using Torrent Suite version 5.10.1, and the annotation of variants was performed by the Ion Reporter Server System v5.16 (https://ionreporter.thermofisher.com). A threshold of at least 20 mutated reads and an allelic frequency of 10% was used to perform mutation calling. Additionally, alignments were visually confirmed by the genome Browse software v3.0. Non-synonymous, insertions/deletions (indels) and mononucleotide variants (MNVs) located in splicesite_5′, splicesite_3′ region that produce missense and nonsense mutations, non-frameshift/frameshift insertion were considered.

The *ROS1* gene status was also assessed by fluorescence *in situ* hybridization (FISH) analysis using ZytoLight ^®^ SPEC ROS1 Dual Color Break Apart Probe (ZytoVision GmbH, Bremerhaven, Germany).

## Results

### Case Report 1

On July 2008, a 59-year-old man, ex-smoker (45 packs/year), underwent upper right lung lobectomy and regional lymph adenectomy with the diagnosis of stage I (pT2, pN0) lung adenocarcinoma, solid pattern. On July 2020, the chest CT scan revealed the presence of an upper left lung lobe and two lower left lung lobe lesions. On August 2020, the patient underwent a wedge resection of the upper and the lower lung lobe**s** lesions with N1 and N2 nodal sampling.

**FIGURE 1:**
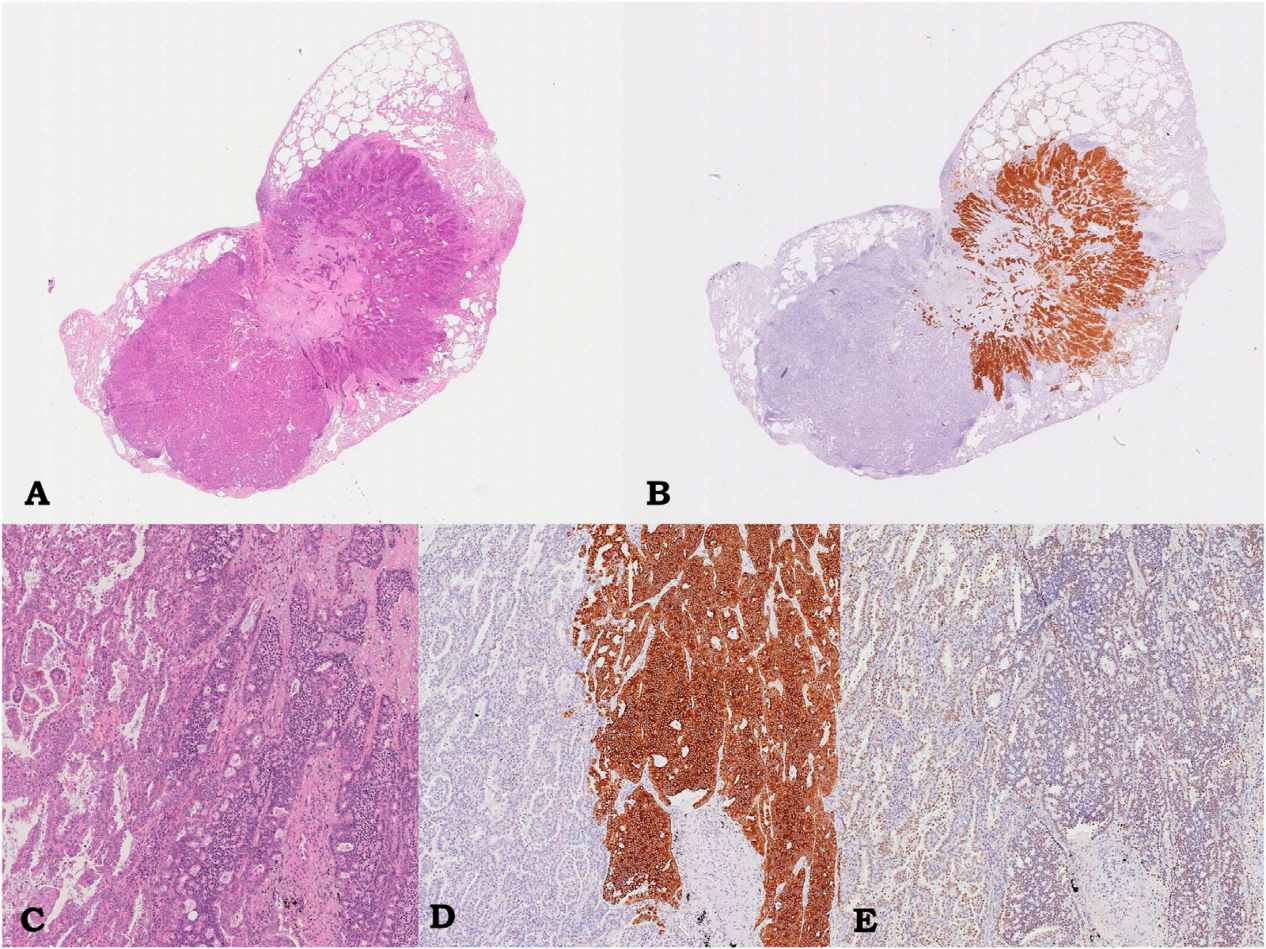
Case 1, pulmonary lesion. Intraparenchymal neoplasm consisting of glandular component corresponding to lepidic-papillary pulmonary adenocarcinoma, on the left, and of adhered trabecular proliferation of monomorphic epithelioid cells corresponding to atypical carcinoid, on the right; hematoxylin/eosin, 0.5x **(A)** and 5.5x **(C)**. Immunostaining showing diffuse and strong immunoreactivity for Synaptophysin only in the carcinoid component **(**on the right, 5.5x; **B, D)** and immunoreactivity for TTF-1 in both component **(**5.5x, **D)**.

Gross examination of the surgical specimens of the first atypical pulmonary resection of left inferior lobe revealed an Intraparenchymal, peripheral, solid, yellow-white lesion measuring 1.4 cm in greatest dimension. Histological assessment identified a well-circumscribed lesion composed of two different morphological components, tightly adhered but not intermingled each other. The first component represented about 60% of the whole neoplasm and was characterized by a solid and trabecular proliferation of polygonal-shaped uniform tumor cells, with nuclei with finely granular chromatin and inconspicuocus nucleoli, consistent with carcinoid. Four mitosis/2 mm^2^ were identified, without tumoral necrosis. The second component, which represented about 40% of whole neoplasm, showed a main lepidic, non-mucinous pattern with secondary papillary architecture, corresponding to a lepidic-papillary pattern PA. Immunoreactivity for chromogranin A, synaptophysin, TTF-1, and pan-cytokeratins AE1–3 was documented in carcinoid component, while adenocarcinomatous component was positive only for TTF-1 and cytokeratins (Figure 1). CK7 immunoreactivity was selectively documented in the adenocarcinomatous component. A final diagnosis of “combined pulmonary adenocarcinoma with atypical carcinoid” was made. No immunoreactivity for ALK and ROS1 was documented in both components. TPS for PD-L1 was <1% in both components. Both two other lesions on atypical pulmonary resections of the upper left lobe and inferior left lobe showed intraparenchymal neoplasm of 1.8 and 1.5 cm, respectively, corresponding both to PA with a main solid pattern and lepidic, non-mucinous second pattern. A positivity ROS1 score of 2+ with gene rearrangement on FISH was documented in the upper pulmonary lobe neoplasia. No immunoreactivity for ALK and a PD-L1 TPS of 1%–49% were observed. No immunoreactivity for ALK and ROS1 with PD-L1 TPS of >50% were documented in the lower lobe neoplasia. No pleural invasion was observed. No lymph nodes metastases were found. The final UICC 2017 stage pT4 (m-3) (PL0) pN0 was assigned.

**FIGURE 2:**
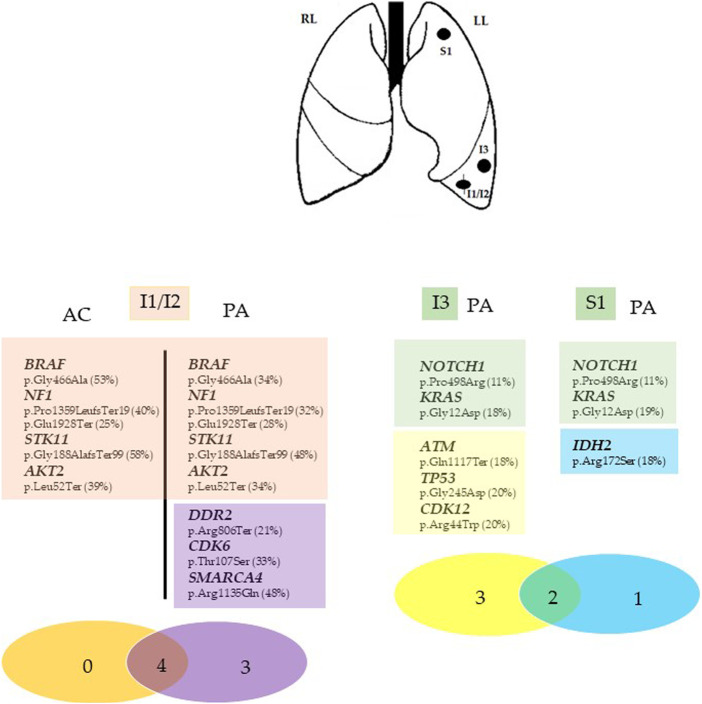
Summary of the genomic findings of Case 1. Genes with mutations identified in each tumor entity of the inferior and superior left lung (I1/I2, I3, and S1) are listed as symbol and aminoacidic changes (% mutant allele fraction). The Venn diagram was used to show the shared mutations (red circle in I1/I2 nodule and green circle in I3 and S1 nodules) and exclusive mutations for each tumor fraction. The different numbers represent the somatic mutations in the corresponding samples, whereas the number in the overlapped regions red (I1/I2) and green (I3 and S1) are ubiquitous somatic mutations shared by the two concurrent tumor components (I1/I2) and by the two isolated adenocarcinoma nodules (I3 and S1) in the same patient. I1/I2, mixedcarcinoid/adenocarcinoma tumor; I3, inferior adenocarcinoma tumor, S1, superior adenocarcinoma tumor. Asterisks (*) indicate the different mutation in the same gene. LL, Left lung; RL, right lung. AC, atypical carcinoid; PA, pulmonary adenocarcinoma.

Targeted NGS analysis on Ion Torrent NGS platform using the Ocav3 panel was performed on DNA and RNA extracted from each microdissected histological components of the primary mixed neoplasm (I1/I2) and in the other two adenocarcinomas located in the inferior (I3) and superior (S1) left pulmonary lobes. Summary of genetic results are shown in Figure 2 **.** Among a total of 161 investigated genes included in the NGS panel, both common and private somatic genes mutations were identified in each entity (Figure 2). The adenocarcinoma I1 and carcinoid I2 components of the mixed neoplasm shared the same mutations in *BRAF* (p.Gly466Ala), *NF1* (p.Pro1359LeufsTer19 and p.Glu1928Ter), *STK11* (p.Gly188AlafsTer99), and *AKT2* (p.Leu52Ter) genes. Four additional somatic mutations were detected in the *DDR2* (p.Arg806Ter), *CDK6* (p.Thr107Ser), and *SMARCA4* gene (p.Arg1135Gln) were identified in the adenocarcinoma component, whereas no specific mutations were identified in the carcinoid component. A different genetic profile emerged from the molecular analysis of the two additional adenocarcinoma nodules I3 and S1, lacking those somatic mutations identified in the mixed nodules, but shared two somatic missense mutations in the *KRAS* (p.Gly12Asp) and *NOTCH1* (p.Pro498Arg). In addition, the I3 adenocarcinoma showed a specific, somatic mutations in *ATM* (p.Gln1117Ter), *TP53* (p.Gly245Asp), and *CDK12* (p.Arg44Trp) genes, whereas the S1 adenocarcinoma showed a specific, somatic, missense mutations in *IDH2* gene (p.Arg172Ser).

Since November 2020, considering the ROS1 positivity, the patient started crizotinib, which is still ongoing, reporting a stable disease.

### Case Report 2

On October 2016, a 66-year-old woman, never smoker, underwent upper left lung lobectomy and regional lymph adenectomy, for a pulmonary mass discovered on radiological examination for shoulder pain present for a long time.

**FIGURE 3:**
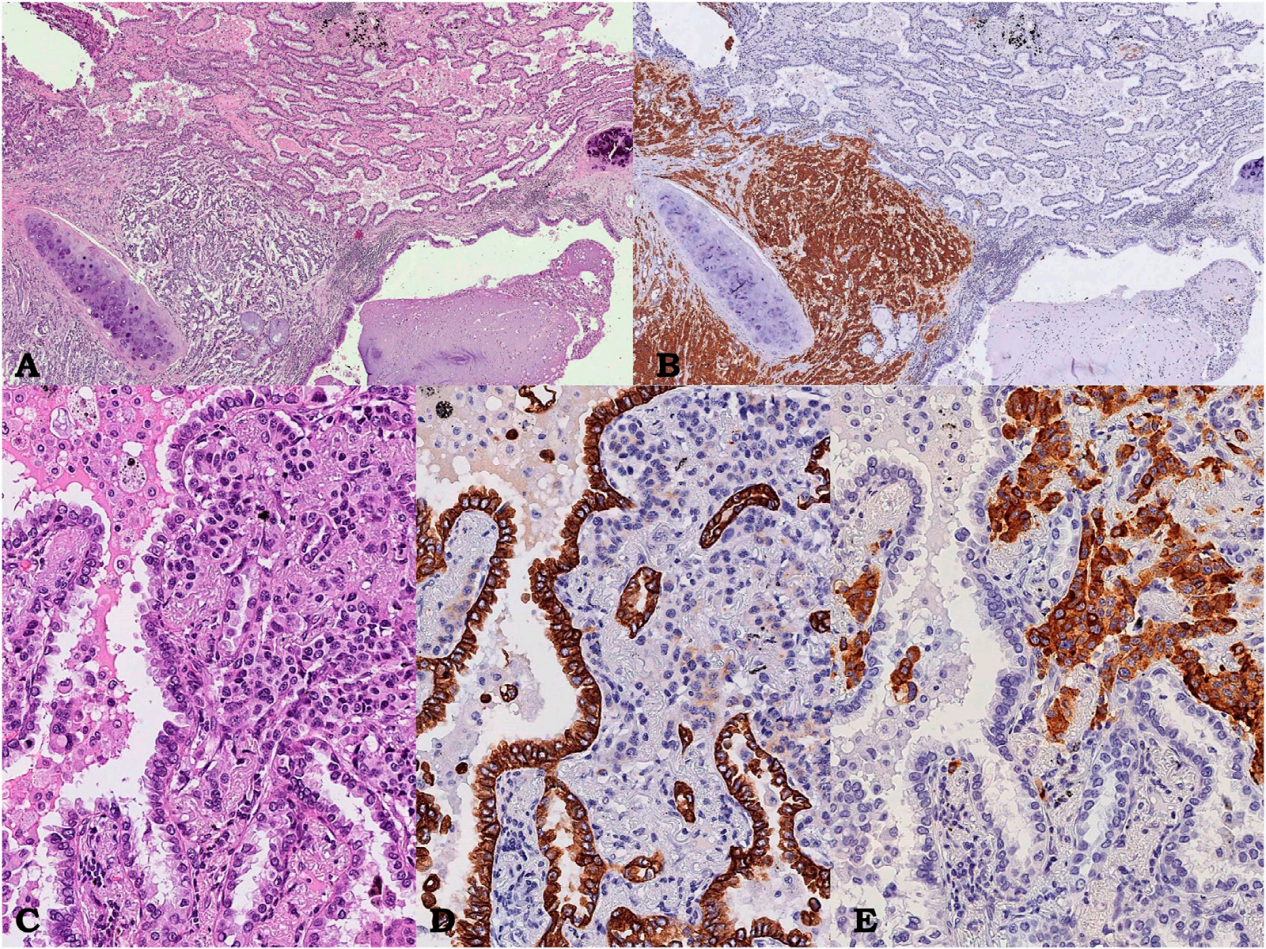
Case 2, pulmonary neoplasm. Intraparenchymal neoplasm showing a glandular component corresponding to an acinar pulmonary adenocarcinoma on the right intermingled with an organoid proliferation of polygonal shaped uniform tumor cells on the left, near bronchus wall, corresponding to atypical carcinoid; hematoxylin/eosin, 3.5x **(A)**. Immunostaining showing diffuse and strong immunoreactivity for Synaptofisin only in the carcinoid component **(**on the left, 3.5x; **B)**, At higher power field, composite neoplasm **(**hematoxylin/eosin, **C**, 15x**)** showing diffuse and strong immunoreactivity for CK7 only in the adenocarcinomatous **(D** 15x**)** and chromogranin only in the neuroendocrine component **(E**, 15x**)**, respectively.

**FIGURE 4:**
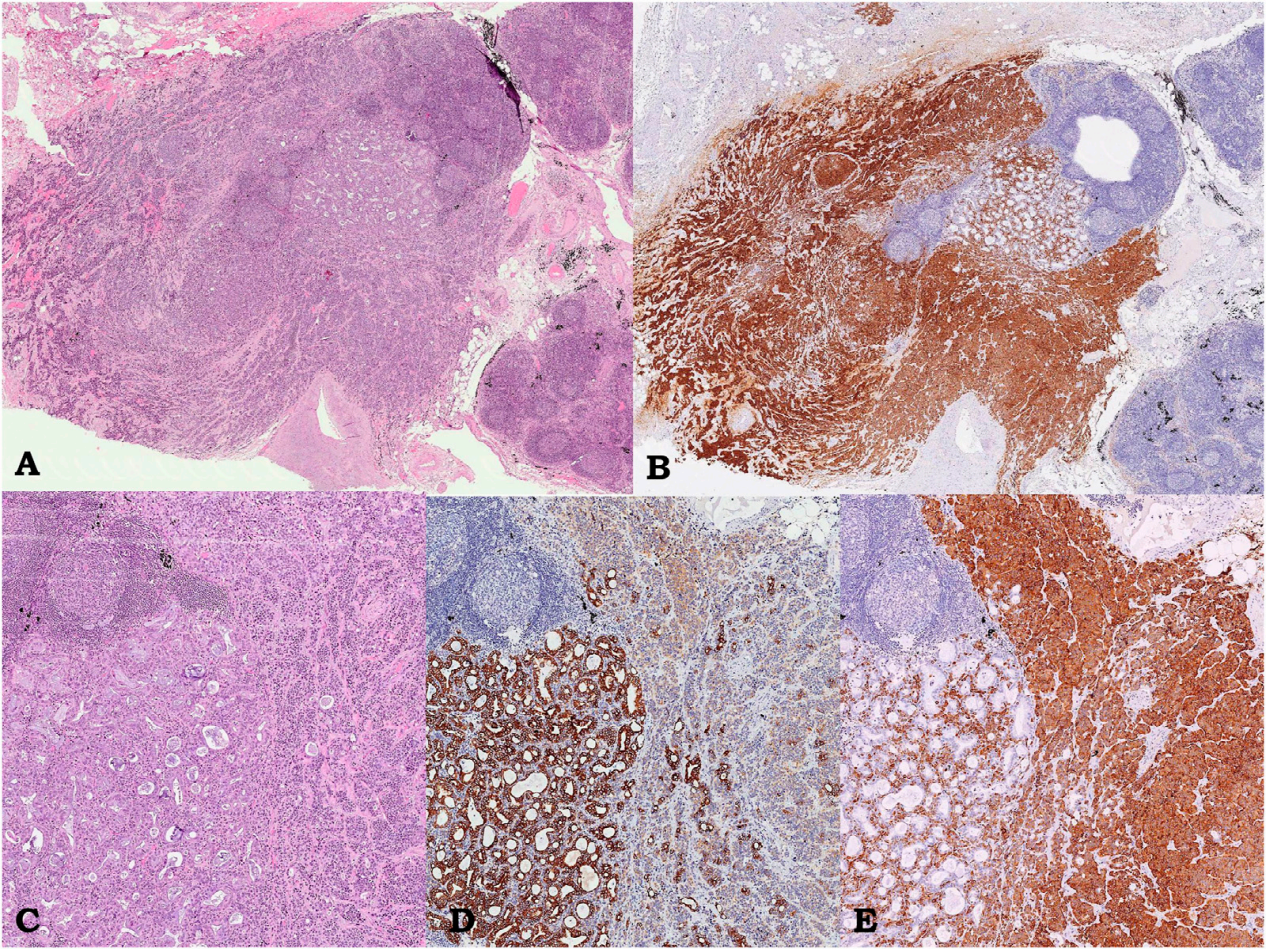
Case 2, lymph node metastasis. Lymph node showing adenocarcinoma component intermingled with carcinoid; hematoxylin/eosin, 3.5× **(A)**. Immunostaining showing diffuse and strong immunoreactivity for Synaptofisin only in the carcinoid component **(**on the left, 3.5×; **B)**. At higher power field, composite neoplasm **(**hematoxylin/eosin, **C**, 4×**)** showing diffuse and strong immunoreactivity for CK7 only in the adenocarcinomatous **(D**, 4×**)** and chromogranin only in the neuroendocrine component **(E**, 4×**)**, respectively.

The gross examination of the surgical specimens showed an intraparenchymal, subpleural, whitish mass, measuring 8.5 cm in greatest dimension. Histologically, a well-circumscribed neoplasia composed of two different morphological components, which were separated in some areas and intermingled in others, was documented. The first component represented about 70% of the tumor burden and consisted of an organoid proliferation of polygonal, shaped uniform tumor cells with nuclei with finely granular chromatin and inconspicuous nucleoli, consistent with carcinoid. Four mitosis/2 mm^2^ and diffuse, punctate necrosis were identified. The second component, representing about 30% of the tumor burden, showed a glandular architecture corresponding to acinar pattern of PA. Neoplastic infiltration of parietal pleura was documented (PL3). Immunoreactivity for chromogranin A, synaptophysin, TTF-1, and pan-cytokeratins (AE1-3 clone) was observed in carcinoid component, while glandular component was positive only for TTF-1 and cytokeratins; cytokeratin 7 was selectively expressed in the adenocarcinomatous component (Figure 3). Metastatis constituted by both neuroendocrine and non-neuroendocrine component was found in four out of six hilar lymph nodes, showing the same immunoreactivity in different neoplastic areas such as primitive lesion (Figure 4). Immunoreactivity for ALK and ROS1 resulted negative in both component, in primitive tumor and in lymph nodal metastasis. TPS for PD-L1 was <1% in both component, in primitive tumor and in lymph nodal metastasis. A final diagnosis of “combined pulmonary adenocarcinoma with atypical carcinoid” was made with stage pT3 (PL3) pN2 sec UICC 2017. Each neoplastic component in both primitive and metastatic lesions was individually microdissected from unstained FFPE slides for DNA and RNA extraction. Targeted NGS analysis on Ion Torrent NGS platform was performed using the Ocav3 panel.

**FIGURE 5:**
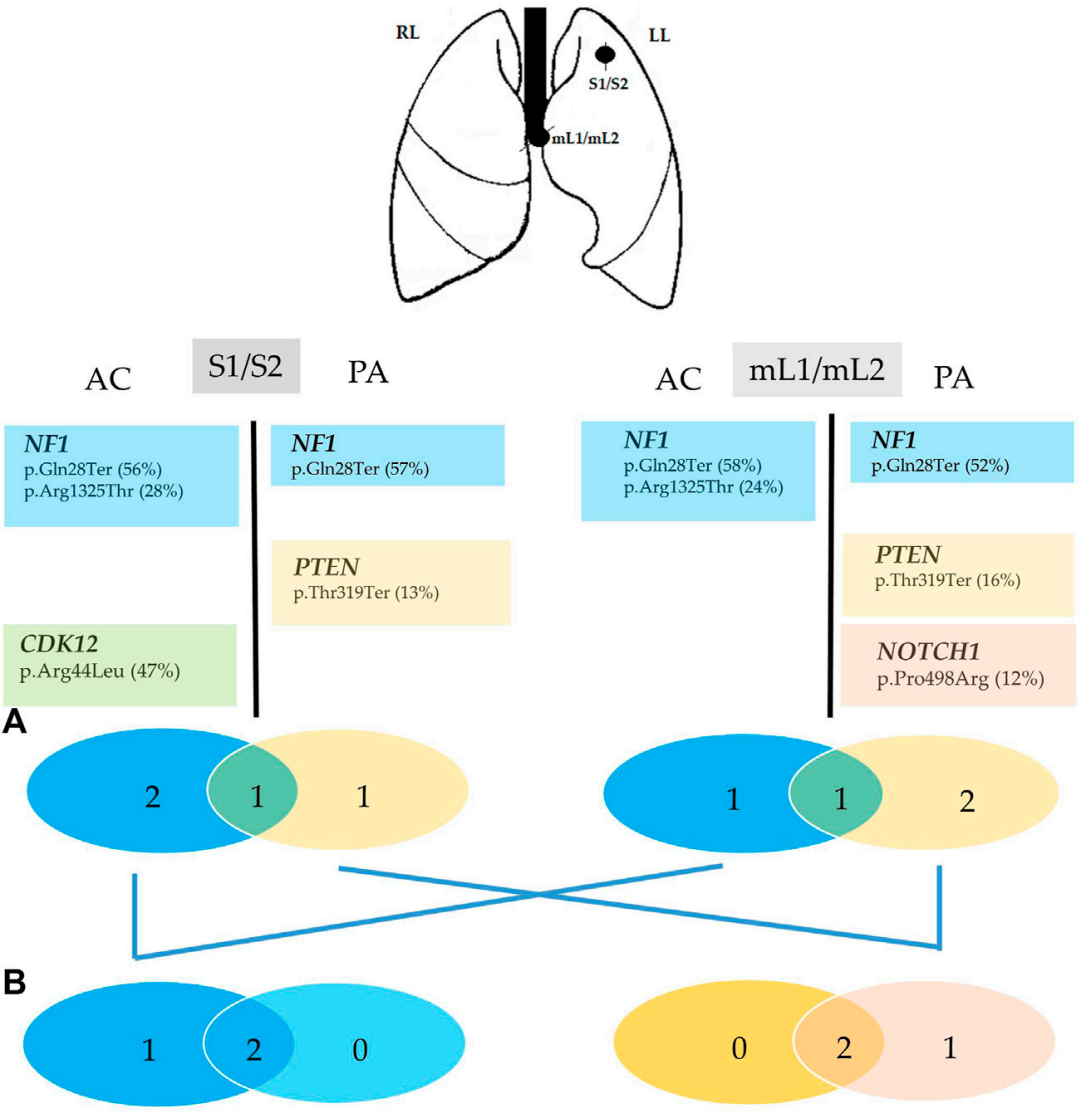
Summary of the genomic findings of Case 2. Genes with mutations identified in each tumor entity of the superior left lung (S1/S2) and hilar lymphnode metastasis are listed as symbol and aminoacidic changes (% mutant allele fraction). The Venn diagrams **(A)** were used to show the relationship of somatic mutations between the S1 (blue circle) and S2 (yellow circle), nL1 (blue circle) and mL2 (yellow circle) in the patient. The number represents the somatic mutations identified in the corresponding samples, and the numbers in the overlapped green regions are the ubiquitous somatic mutations between the two concurrent tumor components (S1/S2, mL1/mL2) in the same patient. The Venn diagrams **(B)** were used to show the relationship of somatic mutations between the carcinoid component of S1 and between the adenocarcinoma component of S2 and mL2. The numbers represent the somatic mutations identified in the corresponding samples, and the numbers in the overlapped regions are ubiquitous somatic mutations shared by the components with the same histology in primary and metastatic sites.S1/S2, mixed carcinoid/adenocarcinoma tumor of the superior lung; mL1/mL2, mixed carcinoid/adenocarcinoma hilar lymphnode metatsasis. LL, left lung; RL, right lung. AC, atypicalcarcinoid; PA, pulmonary adenocarcinoma.

Among the 161 investigated genes, both common and private genes mutations were identified in each investigated entity of primary and metastatic sites (Figure 5). No shared point mutations, CNVs, or gene fusions were identified in both adenocarcinoma and carcinoid components from any sites (S1/S2 and mL1/mL2). By contrast, one somatic mutation in the *PTEN* gene (p.Thr319Ter) was detected specifically in the adenocarcinoma components of both primary and metastatic sites (S2 and mL2), whereas the carcinoid component of both sites shared one somatic mutation in the NF1 gene (p.Arg1325Thr). Primary carcinoid S1 showed one private somatic mutation in the *CDK12* gene (p.Arg44Leu), whereas the adenocarcinoma component of metastatic site showed a private mutation in the *NOTCH1* gene (p.Pro498Arg).

On December 2016, she progressed due to the onset of osteoblastic bone lesions detected by TAC with a negative octreoscan performed on January 2017. In February 2017, cisplatin plus pemetrexed regimen started for six cycles, reporting a partial response after four cycles. In November 2017, due to a massive bone metastasis with bone marrow infiltration, the patient died.

## Discussion

The WHO classification of thoracic tumors defines combined pulmonary neoplasms as an admixture of SCLC and LCNEC or as a coexistence of NSCLC with a carcinomatous neuroendocrine component (WHO, 2021). Rare cases of pulmonary carcinoids combined with a non-carcinoid component have been described, but these tumor types are not yet included in the current WHO classification as a specific entity (La Rosa et al., 2018; Olofson and Tafe, 2018). The coexistence of neuroendocrine and non-neuroendocrine neoplasms is a well-known phenomenon, and available molecular data published point towards a common origin of both components, thus supporting the hypothesis of a monoclonal neoplastic proliferation modality (La Rosa et al., 2018). Molecular studies on colorectal neuroendocrine/non-neuroendocrine carcinomas clearly suggest that the neoplastic proliferations arise from a single (monoclonal) non-neuroendocrine precursor, following the acquisition of one non-neuroendocrine profile that progress towards a more malignant neuroendocrine phenotype, through epigenetic, transcriptional, or translational events (Jesinghaus et al., 2017). Due to their exceptional rarity, very few molecular data supporting this hypothesis are available for mixed lung neoplasms with epithelial carcinomatous component combined with a well-differentiated neuroendocrine component (La Rosa et al., 2018; Olofson and Tafe, 2018).

Specifically, only two cases of combined NSCLC, squamous histotype, and carcinoid (Okazaki et al., 2015; Owens and Fraire, 2011) and two cases of combined NSCLC, adenocarcinoma histotype with typical carcinoid were reported to date (Sen and Borczuk, 1998; Abbi et al., 2014). Unfortunately, none of these cases was investigated at the molecular level, and their genetic profiles are unknown. More recently, two additional cases of combined lung atypical carcinoid and adenocarcinoma were described with a detailed histological and molecular characterization, the last consisting of a common mutational profile of both components, based on NGS panels (Olofson and Tafe, 2018; La Rosa et al., 2018). Specifically, in the first case a *BRAF p.Val600Glu* mutation in both adenocarcinoma and carcinoid components was documented (Olofson and Tafe, 2018). In the second case, the same missense mutations in *KRAS* (p.Gly13Asp), *PAPPA2* (p.Arg901Leu), and *NF1* (p.Val2106Phe) genes were described in both neoplastic components. Moreover, an additional somatic mutation was also detected in neuroendocrine components of both primary and metastatic sites, while a missense mutation in *SMARCA4* gene (p.Pro171Leu) was identified only in the adenocarcinoma component of primary site (Table 1) (La Rosa et al., 2018).

**TABLE 1:** Clinical and pathological features of reported combined pulmonary tumors.

References	NE component	NSCLC histotype	Pathological stadiation	Smoking habit (p/y)	Prognosis	Molecular profile
Okazaki et al. Okazaki et al. (2015)	AC	SqCC	pT3pN0cM0	Former smoker (75 p/y)	PD 9m death 21m	No
Owens et al. Owens and Fraire (2011)	TC	SqCC	T2N2M0	Former smoker (15 p/y)	nr	No
Sen et al. Sen and Borczuk (1998)	TC	PA	pT1pN1cM0	Current smoker (45 p/y)	nr	No
Abbi et al. Abbi et al. (2014)	TC	PA	T1aN0m0 (PA) pT2N0M0 (TC)	Former smoker (50 p/y)	nr	No
Olofson et al. Olofson and Tafe (2018)	AC	PA	pT1apNxcM0	Former smoker (43 p/y)	Alive*	Yes
La Rosa et al. La Rosa et al. (2018)	AC	PA	pT2apN2cM0	Former smoker (nr p/y)	PD 18m Alive 26m	Yes
Parente et al. (present work, Case 1)	AC	PA	pT4(m-3)pN0M0	Former smoker (45 p/y)	SD 14m Alive 14m	yes
Parente et al. (present work, Case 2)	AC	PA	pT3pN2M0	Never smoker	PD 2m Death 13m	Yes

AC, atypical carcinoid; TC, typical carcinoid; PA, pulmonary adenocarcinoma; NE, neuroendocrine; SqCC, squamous cell carcinoma; NSCLC, non-small cell lung cancer; p/y, pack/year; SD, stable disease; PD, progression of disease; m, months; nr, not reported

*no follow-up time reported.

Our results expand the present knowledge about the hypothesis of a clonal origin of these mixed lung tumors and the ability of each histological component to enhance the metastatic process. In Case 1, the molecular profiling revealed both common and private somatic mutations in multiple genes in both neuroendocrine and non-neuroendocrine components, totally different from those identified in the two synchronous, non-mixed lung adenocarcinoma (Table 2). To note, we found common mutations in both carcinoid and adenocarcinoma components of Case 1 in *BRAF* and *STK11* genes that are rarely mutated in primary lung carcinoid (Armengol et al., 2015; Clinical Lung Cancer Genome Project (CLCGP), 2013). In addition, a mutation in the chromatin-remodeling *SMARCA4* gene was identified in the adenocarcinoma component, frequently associated with pure carcinoid histology (Simbolo et al., 2017).

**TABLE 2:** Molecular profile of published combined pulmonary adenocarcinoma and atypical carcinoids of lung primary site of neoplasms.

Reference	Mutated genes		Type of mutation (aa change)		
	NE (AC) component	NSCLC (PA) component	NE (AC) component	NSCLC (PA) component	
Olofson et al. Olofson and Tafe (2018)	***BRAF***	***BRAF***	**p.Val600Glu**	**p.Val600Glu**	
La Rosa et al. La Rosa et al. (2018)	***KRAS***	***KRAS PAPPA2 NF1** SMARCA4*	**p.Gly13Asp p.Arg901Leu p.Val2106Phe**	**p.Gly13Asp p.Arg901Leu p.Val2106Phe** p.Pro171Leu	
	***PAPPA2***				
	***NF1***				
*Present work*	***BRAF***	***BRAF***, ***NF1***, ***NF1***, ***STK11***, ***AKT2***	**p.Gly466Ala, p.Pro1359LeufsTer19, p.Glu1928Ter, p.Gly188AlafsTer99, p.Leu52Ter**, p.Arg806Ter, p.Thr107Ser, p.Arg1135Gln	**p.Gly466Ala, p.Pro1359LeufsTer19, p.Glu1928Ter, p.Gly188AlafsTer99, p.Leu52Ter**	Case 1
	***NF1***				
	***NF1***				
	***STK11***				
	***AKT2***				
	*DDR2*				
	*CDK6*				
	*SMARCA4*				
*Present work*	***NF1***	***NF1***, *PTEN*, *NOTCH1*	**p.Gln28Ter**, p.Arg1325Thr, p.Arg44Leu	**p.Gln28Ter**, p.Thr319Ter, p.Pro498Arg	Case 2
	*NF1*				
	*CDK12*				

AC, atypical carcinoid; PA, pulmonary adenocarcinoma; m, months; NE, neuroendocrine; NSCLC, non-small cell lung cancer; aa change, aminoacidic change.

Shared gene mutations are highlighted in bold.

Molecular and histological characterization of Case 2 gave additional information in supporting the common origin of both neoplastic components. To note, as in the case no.1, we identified in case no.2 non-sense pathogenic mutations in *NF1* gene shared by the two neoplastic components both of primary and of metastatic sites. The *NRF1* mutations, mainly associated with the NSCLC, were reported by La Rosa as shared by both component adenocarcinoma/carcinoid, but their role in the pathogenesis of mixed component remains unknown (Philpott et al., 2017). Finally, the presence of *NOTCH1* private mutation in the adenocarcinoma component only in the primary site, in addition to a common mutation in *PTEN* gene shared by the adenocarcinoma component in primary and nodal metastasis, should support their/its role in inducing metastasis. In this context, transcriptional analysis of such tumors could be also useful to better assess the effect of such mutations and distinguish genetic determinants from passenger mutations, and to assess the existence of intermediate gene expression programs.

Our data add new interesting insight into the understanding of the biology of this unclassified entity, since, if integrated with the previously described, they favor a monoclonal origin of both components, with clinical and therapeutic implications. At this point, some clinical questions arise, such as the following: is there a role for adjuvant chemotherapy? What is the most appropriate regimen for the treatment of advanced disease? Even if the carcinoid component of a mixed tumor should be less aggressive than the NSCLC component, it should be relatively resistant to chemotherapy and radiotherapy, and there is no proven optimal therapy for metastatic unresectable carcinoids tumors (Caplin et al., 2015; Baudin et al., 2021).

Adding more pathological, biomolecular, and clinical information to these rare clinical entities will increase the knowledge and the expertise to manage this specific histotype. In this context, a multicentric study collecting a case series of similar cases is needed to have a better comprehension of molecular mechanisms underlying the pathogenesis of this uncommon tumor entity.

Finally, we propose to classify this rare not-codified entity as “mixed” neoplasm, as yet codified in the 5th edition of Digestive System Tumors in the Mixed Neuroendocrine–Non-neuroendocrine Neoplasm (MiNENs) (WHO, 2019).

## Data Availability

The datasets presented in this study can be found in online repositories. The names of the repository/repositories and accession number(s) can be found below: BioProject PRJNA768323.
