# Supplementary material for: Mixed Pulmonary Adenocarcinoma and Atypical Carcinoid: A Report of Two Cases of a Non-codified Entity With Biological Profile
Source: Front Mol Biosci. 2021 Dec 3;8:784876. doi: 10.3389/fmolb.2021.784876 (PMC8678082; doi:10.3389/fmolb.2021.784876)
Supplement: Supplementary file 1 [file Table1.DOCX]

| Hotspot genes | | | | Full-length genes | | Copy number genes | | Gene fusions (inter- and intragenic) | |
| --- | --- | --- | --- | --- | --- | --- | --- | --- | --- |
| *AKT1/2/3* | *GATA2* | *MYC* | *TERT* | *ARID1A* | *PIK3R1* | *AKT1/2/3* | *NTRK1/2/3* | *AKT2* | *NF1* |
| *ALK* | *GNA11* | *MYCN* | *TOP1* | *ATM* | *PMS2* | *ALK* | *PDGFRA/B* | *ALK* | *NOTCH1/4* |
| *AR* | *GNAQ* | *MYD88* | *U2AF1* | *ATR* | *POLE* | *AXL* | *PIK3CB/A* | *AR* | *NRG1* |
| *ARAF* | *GNAS* | *NFE2L2* | *XPO1* | *ATRX* | *PTCH1* | *AR* | *PPARG* | *AXL* | *NTRK1/2/3* |
| *AXL* | *H3F3A* | *NRAS* |  | *BAP1* | *PTEN* | *BRAF* | *RICTOR* | *BRCA1/2* | *NUTM1* |
| *BRAF* | *HIST1H3B* | *NTRK1/2/3* |  | *BRCA1/2* | *RAD50* | *CCND1/2/3* | *TERT* | *BRAF* | *PDGFRA/B* |
| *BTK* | *HNF1A* | *PDGFRA/B* |  | *CDK12* | *RAD51/B/C/D* | *CCNE1* |  | *CDKN2A* | *PIK3CA* |
| *CBL* | *HRAS* | *PIK3CB/A* |  | *CDKN1B* | *RNF43* | *CDK2/4/6* |  | *EGFR* | *PRKACA/B* |
| *CCND1* | *IDH1/2* | *PPP2R1A* |  | *CDKN2A/B* | *RB1* | *EGFR* |  | *ERBB2/4* | *PTEN* |
| *CDK4/6* | *JAK1/2/3* | *PTPN11* |  | *CHEK1* | *SETD2* | *ERBB2* |  | *ERG* | *PPARG* |
| *CHEK2* | *KDR* | *RAC1* |  | *CREBBP* | *SLX4* | *ESR1* |  | *ESR1* | *RAD51B* |
| *CSF1R* | *KIT* | *RAF1* |  | *FANCA* | *SMARCA4* | *FGF19/3* |  | *ETV1/4/5* | *RAF1* |
| *CTNNB1* | *KNSTRN* | *RET* |  | *FANCD2* | *SMARCB1* | *FGFR1/2/3/4* |  | *FGFR1* | *RB1* |
| *DDR2* | *KRAS* | *RHEB* |  | *FANCI* | *STK11* | *FLT3* |  | *FGFR2/3* | *RELA* |
| *EGFR* | *MAGOH* | *RHOA* |  | *FBXW7* | *TP53* | *IGF1R* |  | *FGR* | *RET* |
| *ERBB2/3/4* | *MAP2K1/2/4* | *ROS1* |  | *MLH1* | *TSC1/2* | *KIT* |  | *FLT3* | *ROS1* |
| *ERCC2* | *MAPK1* | *SF3B1* |  | *MRE11* |  | *KRAS* |  | *JAK2* | *RSPO2/3* |
| *ESR1* | *MAX* | *SMAD4* |  | *MSH6/2* |  | *MDM2/4* |  | *KRAS* | *TERT* |
| *EZH2* | *MDM4* | *SMO* |  | *NBN* |  | *MET* |  | *MDM4* |  |
| *FGFR1/2/3/4* | *MED12* | *SPOP* |  | *NF1/2* |  | *MYC* |  | *MET* |  |
| *FLT3* | *MET* | *SRC* |  | *NOTCH1/2/3* |  | *MYCL* |  | *MYB* |  |
| *FOXL2* | *MTOR* | *STAT3* |  | *PALB2* |  | *MYCN* |  | *MYBL1* |  |

**Table S1**. List of target genes included in the Oncomine Comprehensive Assay v3.
